# Genetic literacy assessment among medical students using the Indonesian adaptation of the University of North Carolina Genomic Knowledge Scale

**DOI:** 10.1007/s12687-026-00940-5

**Published:** 2026-09-16

**Authors:** Rahmat Azhari Kemal, Rahma Dhani, Arya Marganda Simanjuntak, Arimbi Indhira Rafles, Hana Xaviera Triani, Triyanda Miftahur Rahmi, Vinndi Azriel Akbar, Firdaus, Bayu Fajar Pratama, Zulharman

**Affiliations:** 1 Department of Medical Biology, Faculty of Medicine, Universitas Riau, Pekanbaru, Indonesia; 2 Division of Evolution, Infection and Genomics, School of Biological Sciences, Faculty of Biology, Medicine and Health, University of Manchester, Manchester, UK; 3 Faculty of Medicine, Universitas Riau, Pekanbaru, Indonesia; 4 Department of Medical Education, Faculty of Medicine, Universitas Riau, Pekanbaru, Indonesia; 5 Department of Physiology, Faculty of Medicine, Universitas Riau, Pekanbaru, Indonesia

**Keywords:** Genetic literacy, Medical students, Questionnaire, Translation, Validation

## Abstract

**Background:**

Implementation of personalized medicine requires greater genetic literacy among current and future healthcare workers. A standardized Indonesian-language genetic literacy questionnaire is essential for assessment.

**Objective:**

To translate the University of North Carolina Genomic Knowledge Scale (UNC-GKS) genetic literacy questionnaire into Indonesian language, assess its qualitative content validity and internal consistency, and utilized it to measure genetic literacy among Indonesian medical students.

**Methods:**

Following expert panel review and back-translation for translational accuracy and conceptual appropriateness, the questionnaire was pre-tested among 13 preclinical medical students at a public university for comprehensibility and among a separate group of 34 students for internal consistency. The Indonesian UNC-GKS questionnaire was then administered to 486 medical students from a public university comprising 228 preclinical medical students, 187 clerkships, and 71 residents.

**Results:**

The Indonesian version of UNC-GKS showed qualitative content validity as well as good reliability (α = 0.809). Among all students, 56.2% demonstrated moderate overall genetic literacy, and only 14.8% demonstrated good overall literacy. Basic genetic concepts were relatively well-understood (54.1% good literacy). On the contrary, gene variant’s effects on health were poorly understood (9.5% good literacy). Inheritance concepts were moderately understood (24.5% good literacy).

**Conclusion:**

The Indonesian UNC-GKS questionnaire has a good internal consistency making it a reliable genetic literacy assessment tool. Using the questionnaire, we observed predominantly moderate genetic literacy levels in medical students. Further psychometric validation will support broader use of the questionnaire in research and curriculum evaluation. Strengthening genetics education in Indonesian medical schools will support the implementation of personalized medicine in Indonesia’s healthcare system.

**Supplementary Information:**

The online version contains supplementary material available at https://doi.org/10.1007/s12687-026-00940-5.

## Background

Advances in genetics, genomics, and genomic-based technologies are becoming increasingly relevant in all areas and layers of modern society. In the field of medicine, genetics and genomics are applied in diverse areas including prenatal testing, personalized medicines, and even direct-to-consumer genetic testing (Carver et al. 2017). These advances facilitate novel diagnostic capabilities and targeted therapeutic strategies. For instance, genomic technologies can aid in diagnosing patients with rare diseases, congenital anomalies, or developmental disorders. Although such diagnosis may not always lead to immediate treatment options, ending the diagnostic odyssey can offer psychological relief and closure to patients and their families (Clark et al. 2025). Furthermore, genetics is increasingly integrated into reproductive technologies, such as pre-implantation genetic screening and non-invasive prenatal testing, to detect those congenital anomalies and developmental disorders (Go and Shim 2024).

Genetic and genomic technologies can be utilized to obtain patient’s genetic information which can be used to tailor a more personalized treatment and preventions. In oncology, genetic testing plays a crucial role in informing treatment choices as well as identifying at-risk family members who may benefit from early screening and preventative interventions (Bedrosian et al. 2024). For breast cancer patients, information regarding *BRCA1* or *BRCA2* status can inform surgical decision-making and may inform chemotherapy or targeted-therapy selection (Dubsky et al. 2024). The genetic information can also inform preventative actions for patients and their family members. Patients who have not undergone bilateral mastectomy should be recommended breast MRI and mammography. Unaffected family members should be recommended to take genetic testing. If a pre-symptomatic individual is identified as a carrier for the pathogenic variant, they can be prescribed preventative action in the form of intensified breast exam surveillance (Dubsky et al. 2024). This example highlights the relevance and importance of genetics in delivering personalized medicine in clinical practice.

To ensure the effective application of genetic and genomic technologies for personalized medicine, medical professionals must possess adequate genetic literacy (Pereza et al. 2022). Genetic literacy is defined as the sufficient knowledge and understanding of genetic principles that enables individuals to comprehend, interpret, and apply genetic information. It can be measured from the familiarity of genetic terminology, clinical skills, and factual knowledge regarding genes and hereditary traits (Abrams et al. 2015). For medical professionals, from general practitioners to specialists, genetic literacy is essential for identifying potential genetic disorders, delivering appropriate care, and facilitating counselling. Deficiencies in this area may lead to misdiagnosis, suboptimal treatments, and unnecessary or inappropriate use of genetic testing (Swandayani et al. 2021). As an example, 90% of non-genetic residents and specialists surveyed in Croatia thought they might not sufficiently recognize patients with genetic disorders (Mladenić et al. 2024). In the Netherlands, medical specialists involved in the care of breast cancer patients did not always refer eligible patients to a family cancer clinic, including some who meet BRCA testing criteria (van Riel et al. 2010).

To the extent of our knowledge, no studies have assessed genetic literacy among medical doctors in Indonesia. However, it has been argued that low levels of knowledge regarding genetics, clinical genetics, and genetic counselling among healthcare workers in the Asia Pacific region, including Indonesia, may present a significant barrier to the advancement of genetic and genomic services in this region (Thong et al. 2018). In Indonesia, patients are rarely referred for genetic testing which may lead to delayed diagnosis or suboptimal treatment. This stems from limited resources and national policy. In the Indonesian Medical Doctor Competency Standard (*Standar Kompetensi Dokter Indonesia*, SKDI), single-gene and chromosomal disorders are categorized as “good to know” while the complex diseases are taught with limited genetics focus (Rujito et al. 2020). This standard translates into minimal genetics content, mainly addressing introductory basic concepts (Renaldi et al. 2020). In addition, clinical genetics is not established as a distinct medical specialty in Indonesia. Genetic counsellor profession has been developed, but it has not yet formally recognized by the Ministry of Health. The limited professionals in medical genetics hinder high-quality genetics curriculum in medical school, which in turn results in medical professionals with limited genetics knowledge (Gardezi et al. 2026). As medical school is the primary setting for acquiring medical genetics knowledge, educating and assessing genetic literacy among medical students is essential. Several studies revealed concerning patterns in Indonesia. For example, a nationwide survey involving 492 Indonesian medical students from various universities found that only 24.59% of participants achieved a sufficient genetic literacy score of above 50% (Rujito et al. 2020). In contrast, another nationwide survey by Swandayani et al. (2021) found higher levels of self-reported familiarity and genetic literacy among 1,003 Indonesian medical students, with the average scores of 5.63 ± 0.96 (out of 7) and 6.37 ± 0.83 (out of 8), respectively.

In order to objectively assess factual knowledge in genetics, standardized and validated tools are crucial. Several questionnaires have been developed to assess genetic literacy, including the Rapid Estimate of Adult Literacy in Genetics (REAL-G) developed by Erby et al. (2008), the Public Understanding and Attitudes towards Genetics and Genomics (PUGGS) developed by Carver et al. (2017), the Genetic Literacy and Attitudes Survey (iGLAS) developed by Chapman et al. (2019), and the University of North Carolina Genomic Knowledge Scale (UNC-GKS) developed by Langer et al. (2017). These questionnaires have demonstrated good validity and reliability. Among them, UNC-GKS is structured into several parts measuring factual knowledge in basic genetic concepts, effects of gene variation on health, and inheritance. These concepts are part of the core competencies in medical genetics as outlined by the Association of Professors of Human and Medical Genetics (Massingham et al. 2022). This property makes UNC-GKS well-suited to assess genetic literacy among medical students and professionals compared to REAL-G that assesses self-reported familiarity or to PUGSS and iGLAS that use less focused or less structured formats.

The absence of standardized or validated genetic literacy assessment tools in the Indonesian language presents a critical gap in the systematic evaluation of genetic literacy in Indonesian medical education. While Swandayani et al. (2021) has previously used an Indonesian REAL-G translation to assesses self-reported familiarity with eight genetic terms, a more comprehensive instrument is needed to evaluate core medical genetics knowledge that can be utilized to inform genetic literacy assessment and guide targeted educational intervention. To address this need, our study aimed to translate the UNC-GKS genetic literacy questionnaire into Indonesian language, assess its qualitative content validity and internal consistency, and utilized it for genetic literacy assessment among Indonesian medical students at various stages of education at a public university.

## Methods

### Questionnaire translation and validation

The genetic literacy questionnaire used in this study was the UNC-GKS (Langer et al. 2017). The questionnaire consists of 25 items separated into four content areas: genes (Q1-6), gene-health relationship (Q7-12), inheritance (13–19), and whole exome sequencing (20–25). Response options in the UNC-GKS questionnaire were “true”, “false”, and “don’t know”. For this study, we used the first three content areas consisting 19 questions on basic genetic concepts, gene-health relationship, and inheritance.

The translation and adaptation process followed the WHO Guidelines on Translation and Adaptation of Instruments (Younan et al. 2019). The process consisted of the following steps:

1. Forward translation. Each questionnaire was translated from English into Indonesian by a research team member proficient in English and knowledgeable in genetics.
2. Expert panel review. The panel consisted of the initial translator, an expert in (medical) genetics and molecular biology, and an expert in instrument development & translation. In this step, the panels reviewed the items to evaluate whether each item adequately addresses core ideas and has good content validity.
3. Back translation. The Indonesian translation was translated back into English by an expert outside of the research team members. The expert had good command of English and expertise in molecular biology. The back-translated questionnaire was compared to the original English version to assess translation validity.
4. Pretesting and cognitive interview. The draft questionnaires were given to 13 medical students from a public university. All students were in their preclinical year, with 3 students in their first year, 3 students in their second year, and 7 students in their third year. The respondents were asked about the following:
  - What the question/statement was about.
  - Could the respondent paraphrase it in their own words.
  - What the respondent thought when they read the terms used in the question/statement.
  - How the respondent chose their answer.
  - Whether there were any terms that were not understood or inappropriate.

After qualitative assessment for content validity via expert panel and student pre-testing, the final adapted questionnaire was tested for internal consistency in a separate sample of 34 medical students of a public university. This number followed the minimum required sample size of 30 for Cronbach’s alpha (Bujang et al. 2024), with additional 10% dropout margin rounding up to 34 students. All students were in their preclinical year, with 12 students in their first year, 11 students in their second year, and 11 students in their third year. Cronbach’s alpha was calculated using SPSS software for each questionnaire.

### Genetic literacy measurement

Based on the satisfactory Cronbach’s alpha, the Indonesian UNC-GKS questionnaire was further utilized in assessing genetic literacy among medical students at an Indonesian public university in 2022. The students were grouped based on educational stage into preclinical students (years 1–3), clerkship students, and resident doctors. First to third year preclinical students were quota-sampled according to the proportion calculated by Slovin’s formula with 5% margin of error and 10% dropout margin. Preclinical students who became respondents at the pretesting and internal reliability stages were excluded to avoid familiarity bias. Fourth/final year pre-clinical students were excluded because at the time of the study, the students had either finished the compulsory course and were either working on their undergraduate final projects or already in their clerkship placement. The total number of students at first to third year were 156, 127, and 147 students, respectively. Using stratified Slovin’s formula, the minimum sample for each year was 83, 67, and 78 students, respectively.

Clerkship students were sampled from the total population calculated by Slovin’s formula with 5% margin of error and 10% dropout margin. From a total cohort of 319 students, minimum sample of 187 clerkship students was required. At the time of the study, the university only had four residency programs: anesthesiology & intensive care (ANIC, 22 students), obstetrics-gynecology (OBGYN, 38 students), pulmonology & respiratory medicine (PRM, 38 students), and surgery (SURG, 13 students). Therefore, total sampling was employed because of the smaller population size of residents.

A link to an online Google form of the questionnaire was sent to the students via their head of class (preclinical), head of cohort (clerkship), or chiefs (residents). The form was opened from October 2022 – February 2023. Informed consent was placed at the first page of the online form prior to any questions. Percentage of correct answers was categorized into good (≥ 75%), moderate (56–74%), and low (≤ 55%) genetic literacy (Budiman and Riyanto 2013). This research has received ethical clearance from the Faculty of Medicine Universitas Riau (No: B/105/UN19.5.1.1.8/UEPKK/2022).

## Results

### Translation process

In this study, the 19-item UNC-GKS questionnaire has been translated from English to Indonesian language. The expert panel and student pre-testing provided evidence for qualitative content validity of the translated questionnaire. In expert panel discussion, the experts supported the relevance, comprehensiveness, and clarity of sections and questions in the questionnaire. However, the experts raised a point on the required background knowledge, suggesting that the students might not be able to answer the questions due to a mismatch between the questions and the coverage or content of genetics in the curriculum. This concern was also raised by the students at the pretesting stage. They reported that they understood the questions/statements, but found the questions difficult to answer because of the low familiarity and/or understanding of genetic terms and concepts. To improve comprehensibility, the students proposed rewording and/or restructuring several questions. For example, an additional explanation in UNC09 was added for better understanding of “*gejala*” (symptoms) (Table S2).

### Internal consistency

Internal consistency was tested among 34 respondents, consisting of 11 third-year students, 11 s-year students, and 12 first-year students. The mean score between different study years was not statistically different (*p* > 0.05). Cronbach’s alpha analysis showed that the Indonesian UNC-GKS questionnaire demonstrated good reliability (α = 0.809). However, stand-alone subsections had varied reliability. Basic concepts (UNC1 – UNC6), gene and health (UNC7 – UNC12), and inheritance (UNC13 – UNC19) had Cronbach’s α of 0.487, 0.781, and 0.506, respectively. Therefore, the Indonesian UNC-GKS is utilized as a single questionnaire consisting of 19 items covering three content areas as opposed to three standalone shorter questionnaires.

### Genetic literacy of Indonesian medical students

We used the Indonesian UNC-GKS to measure genetic literacy in sampled medical students. The questionnaire is available in Table S2. We sampled a total of 486 medical students consisting of 228 preclinical students, 187 clerkship students, and 71 residents (Table 1). The response rate was only calculated for residents because of the initial target of total sampling as opposed to quota sampling of preclinical and clerkship students. We obtained a 64.0% response rate with 71 respondents for a cohort of 111 residents from four existing programs. The response rate was highest from Anesthesiology & Intensive Care (90.9%), followed by Pulmonology & Respiratory Medicine (89.5%) and Surgery (53.8%), while the lowest response rate was from Obstetrics-Gynecology (26.3%). Because of this uneven distribution of residents across programs, no statistical cross-level comparisons with other groups were performed.

**Table 1:** Characteristics of respondents

Characteristics	Sample (*n*)	Population (*N*)	Percentage from population (%)	Percentage from total sample (%)
Preclinical	**228**	**430**	**53.0**	**46.9**
First year	83	156	53.2	17.1
Second year	67	127	52.8	13.9
Third year	78	147	53.1	16.0
Clerkship	**187**	**319**	**58.6**	**38.5**
Residency*	**71**	**111**	**64.0**	**14.6**
ANIC	20	22	90.9	4.1
OBGYN	10	38	26.3	2.1
PRM	34	38	89.5	7.0
SURG	7	13	53.8	1.4

*ANIC: Anesthesiology & Intensive Care; OBGYN: Obstetrics-Gynecology; PRM: Pulmonology & Respiratory Medicine; SURG: Surgery

The overall score indicated a moderate level of genetic literacy (Table 2). Preclinical students had a significantly lower mean total score compared to clerkship students (*p* < 0.05), while no significant differences were observed between other groups. The mean score for basic concepts reflected borderline good literacy and was consistent across all study levels. In contrast, the mean score for the gene-health relationship concepts indicated low literacy with residents tended to score higher than preclinical students. Inheritance concepts were moderately understood, with preclinical students scoring significantly lower than clerkship students (*p* < 0.05).

**Table 2:** Genetic literacy score between study level

Study level	Genetic literacy score (Mean ± SD)			
	Total	Basic concepts	Gene & Health concepts	Inheritance concepts
Preclinical	61.06 ± 12.72	75.07 ± 16.74	48.54 ± 17.36	59.77 ± 21.08
Clerkship	64.11 ± 12.31	75.13 ± 16.25	52.23 ± 19.15	64.86 ± 20.59
Residency	62.49 ± 13.43	72.77 ± 16.72	54.69 ± 17.63	60.36 ± 24.17
Total	62.44 ± 12.72	74.76 ± 16.53	50.86 ± 18.22	61.82 ± 21.46

**Fig. 1:**
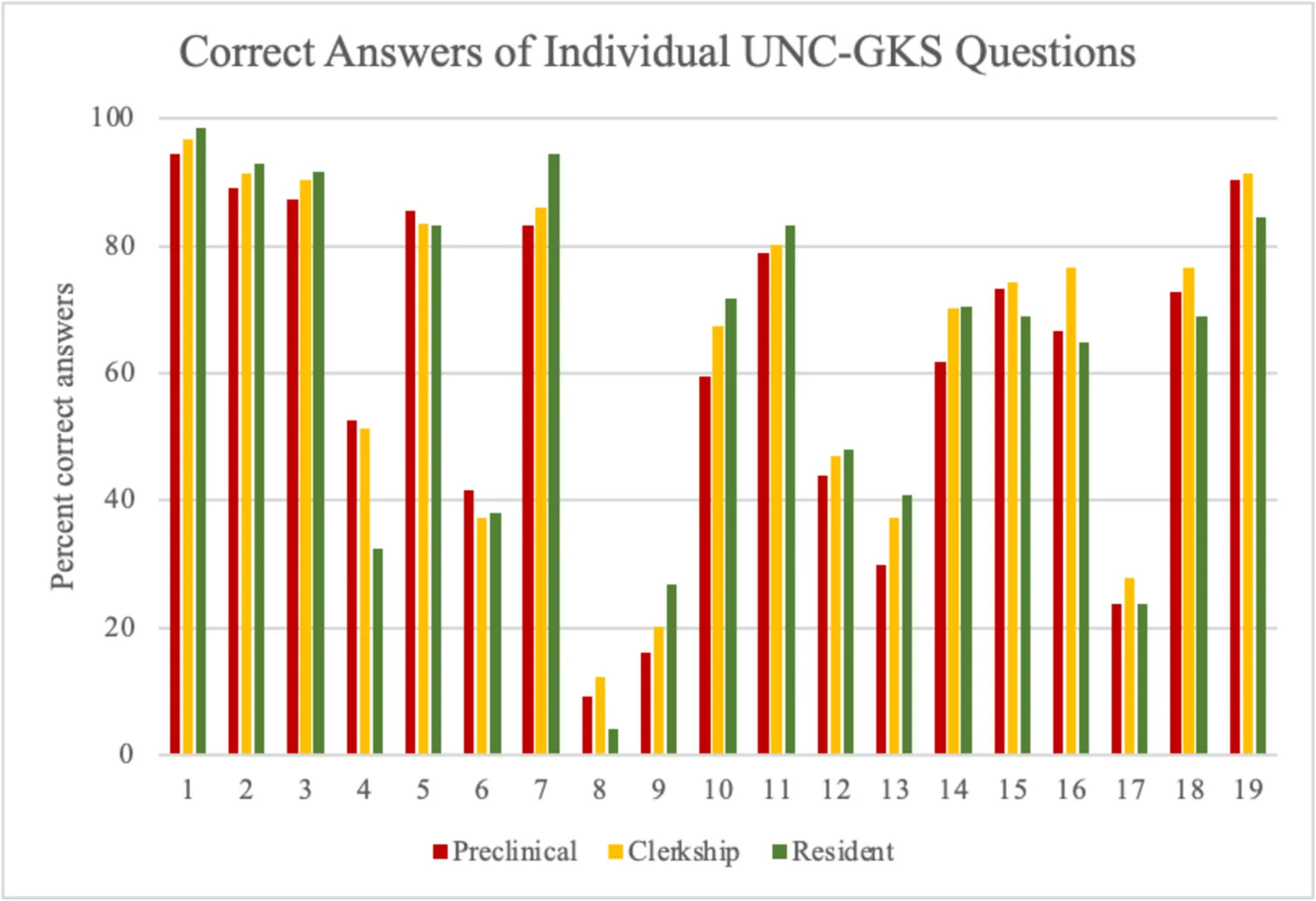
Percentage of correct answers for each individual UNC-GKS showed comparable knowledge between three levels of medical education. No statistical differences were observed between preclinical and clerkship students. Because of the uneven distribution of residency programs, no statistical analysis was conducted against resident subgroup

Categorization of scores resulted in a similar conclusion, with most of the total respondents (56%) demonstrating moderate overall genetic literacy, and only a small subset (15.2%) exhibiting good literacy (Table 3). This distribution pattern was consistent across study levels, with no significant differences observed (*p* > 0.05). Basic genetic concepts were generally well-understood, with more than half of total respondents (54.3%) having a good literacy in this domain. High levels of understanding were observed on gene composition (UNC1), gene functions (UNC2), the genome (UNC3), and DNA building blocks (UNC5) concepts. However, concepts related to gene stability (UNC4) and gene numbers (UNC6) were less well-understood, as shown by a high percentage of students choosing the “don’t know” option (Fig. 1, Table S3). Understanding of the effects of gene variants on health was low, with only 9.7% of respondents having good literacy. The distribution of literacy levels was similar across study levels, with the majority of respondents in each subgroup having low literacy and only a minority showing good literacy (Table 3). Preclinical students had significantly lower knowledge compared to clerkship students (*p* < 0.05). Among the gene-health relationship concepts, only the types of variant effects (UNC7) were well-understood, with over 80% of respondents answering correctly (Fig. 1, Table S3). Other concepts were moderately or poorly understood. Respondents tended to overestimate the proportion of variants that affect an individual’s health (UNC8), which had the lowest correct response rate across all study levels. Additionally, penetrance effects were also overestimated (UNC9). While respondents recognized that different variants have varying effect sizes (UNC10) and that some variants may reduce the risk of certain disorders (UNC11), more than a third of respondents incorrectly believed that the same genetic variant always manifests the same symptoms (UNC12).

The inheritance concept was moderately understood, with 24.9% of respondents exhibiting good literacy (Table 3). While no significant differences were observed in the literacy category between study levels (*p* > 0.05), preclinical students had a significantly lower mean score compared to clerkship students (*p* < 0.05). Among total respondents, most mistakenly believed that genetic disorders are always inherited (UNC13), indicating insufficient understanding of de novo variants (Fig. 1, Table S3). However, respondents correctly recognized that genetic disorders can manifest in only one member of a family (UNC14) and that every individual has a chance for having a child with a genetic disorder (UNC15). Most respondents understood that gene inheritance is not restricted to parent-child pairs of the same sex (UNC16), although the majority incorrectly assumed that physical similarities necessarily correspond to higher genetic similarity (UNC17). The concept of family segregation was partly understood, with majority respondents understanding that if one parent carries a gene variant, their children have a probability of inheriting it (UNC19), but many incorrectly assumed it would be passed down to all children (UNC18).

**Table 3:** Distribution of genetic literacy category among respondents

Section	Literacy	Total	Preclinical	Clerkship	Residency	*p*-value
Overall	Low	141 (29.0%)	70 (30.7%)	49 (26.2%)	22 (31.0%)	0.195
	Moderate	273 (56.2%)	133 (58.3%)	105 (56.1%)	35 (49.3%)	
	Good	72 (14.8%)	25 (11.0%)	33 (17.6%)	14 (19.7%)	
Basic Concept	Low	72 (14.8%)	34 (14.9%)	24 (12.8%)	14 (19.7%)	0.684
	Moderate	151 (31.1%)	68 (29.8%)	62 (33.2%)	21 (29.6%)	
	Good	263 (54.1%)	126 (55.3%)	101 (54.0%)	36 (50.7%)	
Gene & Health	Low	328 (67.5%)	164 (71.9%)	123 (65.8%)	41 (57.7%)	0.022*
	Moderate	112 (23.0%)	52 (22.8%)	39 (20.9%)	21 (29.6%)	
	Good	46 (9.5%)	12 (5.3%)	25 (13.4%)	9 (12.7%)	
Inheritance	Low	135 (27.8%)	70 (30.7%)	43 (23.0%)	22 (31.0%)	0.273
	Moderate	232 (47.7%)	110 (48.2%)	92 (49.2%)	30 (42.3%)	
	Good	119 (24.5%)	48 (21.1%)	52 (27.8%)	19 (26.8%)	

*p-value < 0.05

## Discussion

### Adaptation of the Indonesian UNC-GKS

In personalized medicine, genetic data are integrated into clinical care, leading to more precise diagnosis, treatment selection, and disease prevention strategies (Naithani et al. 2021). Medical professionals must be able to answer genetics-related questions in their daily practice in order to ensure the effective application of genetic and genomic technologies for the public benefit (Cornel and Van El 2017). Assessing this knowledge requires a valid measurement tool. In this study, we successfully translated the UNC-GKS into Indonesian and obtained preliminary evidence supporting its content validity and internal consistency. During the translation process, several items were adjusted, and explanatory phrases were added to improve clarity and contextual understanding. This approach is consistent with methodologies used in other validated adaptations of instruments into Indonesian (Kaligis et al. 2021; Cahyaningsih et al. 2024). The relevance, comprehensiveness, and comprehensibility of the questionnaire supported its content validity. The final Indonesian version of UNC-GKS questionnaire demonstrated good internal consistency, with a Cronbach’s alpha of 0.809, comparable to the original version’s alpha of 0.86 (Langer et al. 2017).

We then utilized the UNC-GKS for further measurement considering its sections being more relevant to the genetic and genomic application in medicine. The basic concepts of genome or gene organization, gene variations, and inheritance are part of the core competencies in medical genetics as outlined by the Association of Professors of Human and Medical Genetics (Massingham et al. 2022). Other genetic literacy questionnaires such as PUGGS (Carver et al. 2017) and iGLAS (Chapman et al. 2019) are not structured into sections that allow a more refined assessment of relevant genetics competencies. In another genetic literacy questionnaire, Genetics Literacy and Reasoning Test (GLRT) developed by Maghfiroh et al. (2025), the questions are mapped into core genetics concepts. However, the concepts and contexts are more general than our medically specific items.

After completion of data collection for the present study in 2023, Hermanto et al. (2024) published their study on genetics knowledge among an Indonesian university’s students using their own 16-item questionnaire. The questionnaire has similar structure to the Indonesian UNC-GKS where it is divided into three subscales, which are basic concepts, heredity, and manifestation of genetic disorder and lifestyle influence. While their third subscale seems to overlap with the Gene & Health section in the Indonesian UNC-GKS, their subscale is more appropriate to assess deterministic view of genetic aspect. Meanwhile, our subscale focuses more on interpreting genetic variants which is an important skill in communicating genetic test results and counselling. In addition, their questionnaire also uses true-false format. The true-false format used in both the Indonesian UNC-GKS and Hermanto questionnaire may be less effective at revealing misconceptions than a multiple-choice format. However, the inclusion of a “don’t know” response option in the Indonesian UNC-GKS reduces the likelihood of guessing and enables the identification of specific items associated with high uncertainty among respondents. This feature could highlight specific areas where targeted educational efforts may be required (Langer et al. 2017). Convergent validity complementarity between the Indonesian UNC-GKS and Hermanto questionnaire could be conducted to further clarify their utilities in genetic literacy assessment in Indonesia.

During pre-testing, the expert panel expressed concerns that medical students might struggle to answer the questions, not because of linguistic issues but because of insufficient exposure to the genetic content in the medical curriculum. This concern was confirmed during cognitive pre-testing, where panel members reported difficulty understanding the genetic terms and concepts. These challenges were reflected in the final result where the majority of respondents (56%) demonstrated moderate overall genetic literacy, while 28.8% exhibited low literacy (Table 3). This distribution was consistent across all study levels (p-value > 0.05). Notably, despite applying a slightly more stringent cut-off for low literacy (≤ 55%) and a limited scope at a single public university, the proportion of students with low literacy remained lower than in the nationwide study by Rujito et al. (2020) where 75.41% scored below 50% measured by an unspecified instrument. Conversely, a national survey by Swandayani et al. (2021) found a higher genetic familiarity and literacy among Indonesian medical students. However, Swandayani et al. (2021) assessed self-reported familiarity of genetic terms using an Indonesian REAL-G translation, as opposed to our factual knowledge assessment using the Indonesian UNC-GKS. The higher terminology familiarity might reflect Indonesia’s academic system where medical school applicants, typically natural science majors in high school, may have increased familiarity with scientific terms. This is supported by an observation at an Indonesian public university, where natural science majors demonstrated higher genetic knowledge than social science majors (Hermanto et al. 2024).

### Genetic literacy among Indonesian medical students

The UNC-GKS has three sections addressing different genetic concepts, which are basic genetic concepts, gene-health relationship, and inheritance. Our study revealed that the highest understanding was in the basic genetic concepts compared to gene-health relationship and inheritance concepts. More than half (54.3%) of respondents had good literacy. Our findings aligned with other studies, where similar high confidence in core genetic-genomic concepts but lower in understanding genetics’ contribution to disease was observed (Seed et al. 2024). However, it is important to note that in both the original and Indonesian versions of UNC-GKS, the genome is stated as “all genetic information”. While technically correct, this wording may fail to identify misconceptions about genome composition. In a small study among Indonesian medical students, more than half of the respondents equated the genome with all genes, rather than all DNA sequences (Kemal et al. 2022). Similar misunderstandings regarding the relationship between genes and DNA was also observed among Indonesian undergraduate students (Hermanto et al. 2024).

Unfortunately, only 9.7% of our respondents had good genetic literacy regarding effects of gene variants on health. Similar to Swandayani et al. (2021), high self-reported familiarity with “variation” did not translate to correct answers (< 10%). Indonesian undergraduate students, even healthcare majors (only 71%), struggled with the healthy-carrier concept, indicating misunderstanding of genetic risk manifestation (Hermanto et al. 2024). Similar trends exist globally. A UK survey by Seed et al. (2024) revealed only half of medical students were confident in understanding genetics’ role in diseases, despite high confidence in basic genetic concepts (95%) and inheritance patterns (90%). Poor knowledge on genetic diseases was also reported in clinical-year medical students in Malaysia (Lin et al. 2022), obstetrics-gynecology, pediatrics, and neurology residents and specialists in Croatia (Mladenić et al. 2024) as well as among general practitioners, gynecologists, and pediatricians in the Netherlands (Baars et al. 2005). On the other hand, the inheritance concept was only moderately understood, with only 24.9% of total respondents having a good literacy level. Similarly, medical students and interns in Saudi Arabia reported lower knowledge on genetic inheritance (Zakariyah et al. 2024). In contrast, 90% of UK medical students reported good confidence in understanding inheritance patterns (Seed et al. 2024). Among primary care physicians and medical specialists, overall knowledge on hereditary breast cancer was good. However, knowledge on breast cancer inheritance was more limited (van Riel et al. 2010; Dekanek et al. 2020). This limited understanding on how genes are inherited in families might be related to limited comprehension of Mendelian concepts. It has been argued that a student’s understanding of Mendelian concepts is essential to support understanding of other inheritance topics (Kaminske et al. 2020). Supporting this, a small study involving 25 Indonesian pre-service biology teachers reported lower Mendelian inheritance scores compared to other genetic topics (Mustofa et al. 2024). In a different Indonesian university, many undergraduate students have difficulties in understanding the Mendelian principles and reconstructing chromosomes based on Mendel’s laws (Maghfiroh et al. 2024). These difficulties might have arisen since high school. Review of Indonesian high school biology textbooks revealed incomplete coverage of genetic concepts and the presence of potential misconceptions (Kusniawati et al. 2025).

Low literacy on genetic variants and inheritance can affect clinical practice. Good knowledge on interpreting genetic variants and inheritance patterns is relevant to clinical practice, especially for communicate genetic test results and providing counseling to not only patients but also their family. Primary care physicians in Hong Kong and Shenzen, China had low genetic knowledge and self-rated confidence in explaining genetic testing results and providing genetic counselling to patients (Yu et al. 2021). A scoping review shows surgeons’ lack of knowledge and confidence in genetic inheritance impacts their comfort with genetic testing and counseling (Mir et al. 2024). To implement personalized medicine effectively, medical doctors require knowledge to interpret genetic test results and identify patients who may benefit from genetic testing. Challenges in accurately interpreting genetic and genomic test results may hinder integration of genetic or genomic information in clinical-decision making such as treatment selection (West and Lovly 2023). Inadequate genetics knowledge may also contribute to failure in obtaining adequate family history, hindering identification and referral to genetic services (Delikurt et al. 2015).

We argue that the lower literacy on understanding variants and inheritance compared to basic concepts is resulted from the current genetics content in the Indonesian medical curriculum. This argument echoes the concern raised by the experts and students during the initial pre-testing. The proportion of genetics content is already minimal and mainly addressing the basic concepts (Renaldi et al. 2020; Rujito et al. 2020). This makes the current curriculum does not equip the future doctors with the basics of genomics and genetic screening (Octavius et al. 2023). Indonesia currently lacks formal genetics or genomics curriculum for medical schools, a challenge shared with other lower-middle income countries (Gardezi et al. 2026). Therefore, it is urgent to develop medical genetics curriculum and learning materials to be integrated into the Indonesian Medical Doctor Competency Standard (*Standar Kompetensi Dokter Indonesia*, SKDI). The Indonesian Medical Council and relevant authorities in Indonesian medical education should develop the curriculum and core competencies for medical doctors in collaboration with the Indonesian Society of Human Genetics. Such approach can be adapted from the Association of Professors of Human and Medical Genetics (APHMG) in developing consensus core medical genetics competencies in the USA (Massingham et al. 2022). Consensus for definition and measurement of genetic literacy is also needed (Daly and Kaphingst 2023; Kaphingst 2023). For the current general practitioners and specialists, online-based continuing medical education programs in genetic or genomic medicine can be developed (Seed et al. 2024). Online training was shown to help surgical oncologists and nurses to have a positive attitude, confidence, and self-efficacy to provide pre-test genetic counselling to breast cancer patients (Bokkers et al. 2023). A Learning Management System-based genetic module for self-paced study can also be utilized (Zulharman et al. 2022). The Indonesian UNC-GKS can serve as the evaluation measure for new curriculums and educational interventions. By introducing a high-quality genetics curriculum both in medical school and as a continuing medical education, we expect Indonesia to have medical professionals with higher genetics knowledge. These genetics-literate medical professionals will be able to utilize genetics and genomics technologies to deliver personalized medicine to their patients (Gardezi et al. 2026).

The Indonesian UNC-GKS appears to be a reliable tool for assessing genetic literacy among medical students. We hope this questionnaire can be utilized in a nationwide genetic literacy measurement. Such assessment can provide a more representative figure to inform the national genetics curriculum in Indonesian medical schools. The questionnaire can also serve as a foundational option in determining consensus definition and measurement of genetic literacy in Indonesia (Daly and Kaphingst 2023; Kaphingst 2023). In addition, further studies are needed to evaluate the questionnaire applicability in other healthcare worker groups such as nurses, pharmacists, and nutritionists, given the potential for gaps in genetic knowledge among those populations, as found in a pilot study of Indonesian nursing students (Kusumaningrum and Erawati 2018). Genetic literacy is a relevant competency for nurses to provide support and guidance to individuals and families undergoing genetic testing (Prows et al. 2014; Calzone et al. 2024). Moreover, as pharmacogenetics and nutrigenetics become more relevant in clinical practice, good genetic literacy is also needed by pharmacists and nutritionists (Rahma et al. 2021; Farhan et al. 2024). Finally, the questionnaire may also be used to assess genetic literacy in the general population, as in the original instrument (Langer et al. 2017).

### Limitations

Our study translated the UNC-GKS into Indonesian and assessed the translation’s internal reliability. Content validity was established through expert-panel evaluation of item relevance, comprehensiveness, and clarity as well as through pilot testing with preclinical medical students to assess comprehensibility. The original questionnaire has been tested for its convergent validity where it has positive correlation with familiarity on genetic terms measured by REAL-G (Langer et al. 2017). Future studies on psychometric evaluations should be conducted. For example, factor analysis to assess construct validity or comparison with questionnaire by Hermanto et al. (2024) to assess convergent validity can be performed.

We initially aimed for total sampling of residents. However, after the initial distribution and two follow-ups, no additional response was obtained. Two programs, ANIC and PRM, had response rates around 90%, while only around half of SURG residents and a quarter of OBGYN residents responded. Because of this uneven response rate and skewed composition, the findings for the residency group should be regarded as exploratory rather than generalized representative. Additionally, although the study site provides general mandatory lectures in molecular biology and genetics to all residency programs, exposure to genetics-related cases during training differs between specialties. Therefore, the genetic literacy levels observed in the combined residency group should be interpreted cautiously.

While our study was limited to 486 medical students from a single institution, we obtained similarly low genetic literacy as observed in a larger nationwide study on medical students from various universities conducted by Rujito et al. (2020). Our study also observed similar pattern across concepts area, with higher literacy in basic concepts but lower literacy in gene-health relationships, observed by Hermanto et al. (2024) among 1596 students from various majors at an Indonesian public university. Taken together, our study provides a relevant snapshot of genetic literacy among Indonesian medical students.

## Conclusions

The Indonesian translation of UNC-GKS genetic literacy questionnaire demonstrated preliminary qualitative content validity and good internal consistency. Using the Indonesian UNC-GKS, we found moderate genetic literacy among medical students at a public university, with good understanding on basic genetic concepts. The more limited knowledge on variant effects and inheritance calls for a revision in the study site’s medical genetics curriculum. Integrating genetic variant interpretation and family segregation in the curriculum is expected to improve the student’s understanding in these domains which will help them in communicating result and providing counselling to their patients in the future. The Indonesian UNC-GKS can be used in multi-center or nationwide studies to thoroughly capture the landscape of genetic literacy among Indonesian medical students. The findings will be able to inform Indonesia’s medical genetics curriculum. The questionnaire can be used to evaluate new curriculums and track improvements. Further validation through psychometric evaluations as well as testing among other healthcare professionals is recommended. Improved genetic literacy among Indonesian medical and healthcare workers could facilitate genetic technology adoption for personalized medicine implementation, potentially leading to better patient care and health outcomes.

## Supplementary Information

Below is the link to the electronic supplementary material.

Supplementary Material 1

## Data Availability

The data that support the findings of this study are available from the authors upon reasonable requests.
