# Supplementary material for: Genetic literacy assessment among medical students using the Indonesian adaptation of the University of North Carolina Genomic Knowledge Scale
Source: J Community Genet. 2026 Sep 16;17(5):108. doi: 10.1007/s12687-026-00940-5 (PMC13582748; doi:10.1007/s12687-026-00940-5)
Supplement: Supplementary file 1 [file 12687_2026_940_MOESM1_ESM.docx]

**GENETIC LITERACY ASSESSMENT AMONG MEDICAL STUDENTS USING THE INDONESIAN ADAPTATION OF THE UNIVERSITY OF NORTH CAROLINA GENOMIC KNOWLEDGE SCALE**

**Supplementary materials**

Table S1. Suggestions from pretesting and cognitive interview

| **Item** | **English Version** | **Translation** | **Pretesting & interview result** | **Note** |
| --- | --- | --- | --- | --- |
| UNC  09 | Everyone who has a harmful gene variant will eventually have symptoms | *Seseorang yang memiliki sebuah varian gen yang berbahaya pasti akan memiliki gejala* | *Seseorang yang memiliki sebuah varian gen yang berbahaya pasti akan memiliki gejala dari varian tersebut* | Providing additional context that the symptoms are the ones from the gene variant |

Table S2. The Indonesian UNC-GKS questionnaire

| Concept | No | Item |
| --- | --- | --- |
| Basic Concepts | UNC1 | Gen tersusun dari DNA |
|  | UNC2 | Gen mempengaruhi kesehatan dengan mempengaruhi protein yang dibuat oleh tubuh |
|  | UNC3 | Seluruh informasi genetik seseorang disebut genom |
|  | UNC4* | Gen-gen seseorang berubah total setiap 7 tahun sekali |
|  | UNC5 | DNA dalam gen terdiri dari empat unit penyusun (A, C, T, dan G) |
|  | UNC6 | Manusia memiliki sekitar 20.000 hingga 25.000 gen |
| Gene Variant and Health | UNC7 | Variasi gen dapat menimbulkan efek positif, efek berbahaya, ataupun tidak berefek bagi kesehatan |
|  | UNC8* | Sebagian besar varian gen akan mempengaruhi kesehatan seseorang |
|  | UNC9* | Seseorang yang memiliki sebuah varian gen yang berbahaya pasti akan memiliki gejala dari varian tersebut |
|  | UNC10 | Sebagian varian gen memiliki efek yang besar bagi kesehatan sementara sebagian yang lain memiliki efek yang kecil |
|  | UNC11 | Beberapa varian gen menurunkan kemungkinan timbulnya suatu kelainan |
|  | UNC12* | Dua orang yang tidak berkerabat namun memiliki varian genetik yang sama akan selalu memiliki gejala yang sama |
| Gene Inheritance | UNC13* | Kelainan genetik selalu diwariskan dari salah satu orang tua |
|  | UNC14* | Jika hanya satu orang dalam suatu keluarga yang memiliki kelainan, kelainan tersebut bukan disebabkan oleh genetik |
|  | UNC15 | Setiap orang berpotensi memiliki anak dengan kelainan genetik |
|  | UNC16* | Anak perempuan mewarisi sebagian besar gennya dari ibunya sedangkan anak laki-laki mewarisi sebagian besar gennya dari bapaknya |
|  | UNC17* | Sepasang ibu dan anak perempuannya yang terlihat mirip memiliki kemiripan genetis yang lebih tinggi dibandingkan sepasang ibu dan anak peremuannya yang tidak terlihat mirip. |
|  | UNC18* | Jika salah satu orang tua memiliki varian genetik yang berbahaya, seluruh anaknya akan mewarisi varian tersebut. |
|  | UNC19 | Jika salah satu orang tua Anda memiliki varian gen, adik atau kakak Anda mungkin juga memiliki varian tersebut |

*The correct answer for the negative questions is “False”.

Table S3. Percentage of correct answer for each UNC-GKS question in different medical study level

| Concept | No | Total (%) | | Preclinical (%) | | Clerkship (%) | | Residency (%) | | p-value |
| --- | --- | --- | --- | --- | --- | --- | --- | --- | --- | --- |
|  |  | Correct | Don’t know | Correct | Don’t know | Correct | Don’t know | Correct | Don’t know |  |
| Basic concept | UNC1 | 95.9 | 0.4 | 94.3 | 0.9 | 96.8 | 0 | 98.6 | 0 | 0.367 |
|  | UNC2 | 90.5 | 4.9 | 89.0 | 5.7 | 91.4 | 4.8 | 93.0 | 2.8 | 0.812 |
|  | UNC3 | 89.1 | 4.1 | 87.3 | 4.4 | 90.4 | 4.3 | 91.5 | 2.8 | 0.732 |
|  | UNC4 | 49.2 | 42.0 | 52.6 | 37.7 | 51.3 | 41.7 | 32.4 | 56.3 | **0.031** |
|  | UNC5 | 84.4 | 7.0 | 85.5 | 7.0 | 83.4 | 5.3 | 83.1 | 11.3 | 0.266 |
|  | UNC6 | 39.5 | 39.1 | 41.7 | 36.8 | 37.4 | 42.2 | 38.0 | 38.0 | 0.805 |
| Gene variant and health | UNC7 | 86.0 | 5.3 | 83.3 | 7.0 | 86.1 | 4.8 | 94.4 | 1.4 | 0.197 |
|  | UNC8 | 9.7 | 4.5 | 9.2 | 5.7 | 12.3 | 3.2 | 4.2 | 4.2 | 0.256 |
|  | UNC9 | 19.3 | 12.8 | 16.2 | 13.6 | 20.3 | 11.8 | 26.8 | 12.7 | 0.372 |
|  | UNC10 | 64.4 | 20.6 | 59.6 | 24.6 | 67.4 | 18.2 | 71.8 | 14.1 | 0.233 |
|  | UNC11 | 80.0 | 10.1 | 78.9 | 11.4 | 80.2 | 9.1 | 83.1 | 8.5 | 0.883 |
|  | UNC12 | 45.7 | 17.7 | 43.9 | 18.0 | 47.1 | 18.2 | 47.9 | 15.5 | 0.928 |
| Inheritance | UNC13 | 34.4 | 1.6 | 29.8 | 2.2 | 37.4 | 1.1 | 40.8 | 1.4 | 0.314 |
|  | UNC14 | 66.3 | 7.2 | 61.8 | 7.5 | 70.1 | 7.5 | 70.4 | 5.6 | 0.356 |
|  | UNC15 | 73.0 | 8.8 | 73.2 | 8.8 | 74.3 | 7.0 | 69.0 | 14.1 | 0.515 |
|  | UNC16 | 70.2 | 10.3 | 66.7 | 13.2 | 76.5 | 7.5 | 64.8 | 8.5 | 0.080 |
|  | UNC17 | 25.3 | 15.8 | 23.7 | 15.8 | 27.8 | 15.5 | 23.9 | 16.9 | 0.898 |
|  | UNC18 | 73.7 | 5.6 | 72.8 | 7.0 | 76.5 | 3.7 | 69.0 | 5.6 | 0.516 |
|  | UNC19 | 89.9 | 5.3 | 90.4 | 6.6 | 91.4 | 4.3 | 84.5 | 4.2 | 0.054 |
